# Principles of miRNA-Target Regulation in Metazoan Models

**DOI:** 10.3390/ijms140816280

**Published:** 2013-08-07

**Authors:** Epaminondas Doxakis

**Affiliations:** Basic Neurosciences Division, Biomedical Research Foundation of the Academy of Athens, Soranou Efesiou 4, Athens 11527, Greece; E-Mail: edoxakis@bioacademy.gr; Tel.: +30-210-6597-479; Fax: +30-210-6597-545

**Keywords:** miR, miR biogenesis, miR targets, miR turnover, isomiR, ceRNA

## Abstract

MicroRNAs (miRs) are key post-transcriptional regulators that silence gene expression by direct base pairing to target sites of RNAs. They have a wide variety of tissue expression patterns and are differentially expressed during development and disease. Their activity and abundance is subject to various levels of control ranging from transcription and biogenesis to miR response elements on RNAs, target cellular levels and miR turnover. This review summarizes and discusses current knowledge on the regulation of miR activity and concludes with novel non-canonical functions that have recently emerged.

## 1. Introduction

Mature microRNAs (miRs) are a class of highly conserved small non-coding RNA molecules, about 22 nucleotides in length, that act to inhibit protein expression by partially hybridizing to complementary sequences, mainly in the 3′ UTR, of target RNA transcripts. Each miR is estimated to regulate multiple functionally-related target mRNAs, and the combinatorial action of miRs is expected to regulate the expression of hundreds of mRNAs. Currently, over 1100 and 1800 miRs have been annotated and categorized in mice and humans, respectively (miRBase 20, [1]). However, these numbers are likely to be inflated by mistakenly identified miRs [2]. In addition, the high rate of miR family turnover in mammals—with many newly emerged miR families being lost soon after their formation—indicate that many more of the truly-identified miRs are likely to have little functional significance [3]. It is now predicted that more than half of human genes are regulated by miRs [4]. miRs have a wide variety of tissue expression patterns and are differentially expressed during development [5–8]. They are deregulated in most human diseases and the profiles they generate carry more diagnostic information than those of mRNAs or proteins [9]. Moreover, the therapeutic potential of miRs, already demonstrated in numerous studies, has further heightened the importance of research that seeks to understand both their mechanism of action and their biological significance [10–13].

This paper aims at reviewing the latest information on miR biogenesis and the factors that determine the efficacy of miR-mediated repression and miR endogenous levels. It concludes with novel atypical functions that stand-out from the canonical repression activity of miRs.

## 2. miR Biogenesis

**Figure 1:**
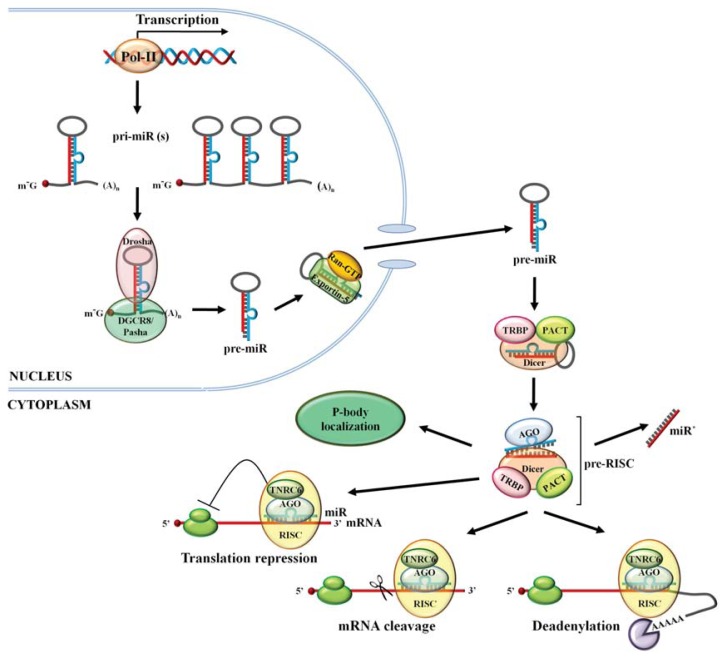
miR biogenesis. Monocistronic or polycistronic miRs are transcribed by RNA polymerase II into long pri-miR transcripts. These pri-miRs are, subsequently, processed by RNase III Drosha complex to ~70 nt pre-miRs that are exported out of the nucleus and into the cytosol by Exportin-5. In the cytoplasm, the RNase III Dicer complex cleaves pre-miRs to double-stranded ~22 nt miRs. One strand is then selected and loaded onto an Argonaute protein, which is part of the miRISC complex. The single-stranded miR then serves as a guide for RNA interference based on the partial complementarity with the various RNA substrates.

miRs are transcribed as part of longer primary transcripts (pri-miRs) by, mainly, RNA polymerase II (Pol II) and only few by RNA polymerase III (Pol III) [14–19]. The majority of miR genes are transcribed from introns of protein-coding genes. The remaining are transcribed as part of long non-coding RNAs that are often arranged as clusters that lead to one pri-miR being subsequently, processed into several mature miRs (Figure 1) [17,20]. Similar to other Pol II transcripts, pri-miRs possess a 5′ 7-methyl-guanosine cap and a 3′ poly (A) tail, the use of which is currently poorly understood. Within pri-miR long transcripts, mature miR sequences form hairpin structures that contain imperfect double-stranded stems of ~30 bp connected by a terminal loop at the top and single-stranded RNA segments at the base [21,22]. In the canonical miR biogenesis pathway, these hairpin structures are recognized in the nucleus and cleaved by a multi-protein microprocessor complex that is composed of two core components, Drosha (a RNase III ribonuclease) and DGCR8 (also known as Pasha in invertebrates which is a double-stranded RNA binding protein). Mechanistically, DGCR8, initially, recognizes the base of the miR hairpin structure and then guides Drosha to cleave the pri-miR at a distance of ~11 bp from the base generating a ~70 nucleotide (nt) hairpin RNA (named precursor miR or pre-miR) with a 2 nt 3′ overhang [21–26]. This 3′ overhang and the double-stranded hairpin structure of the pre-miR are subsequently recognized by exportin-5, which together with its cofactor RAN-GTP, shuttle pre-miR from the nucleus into the cytosol. The hydrolysis of GTP bound to RAN in the cytosol triggers the dissociation of the complex, allowing the pre-miR to bind Dicer, a double-stranded ribonuclease III. Dicer cleaves the pre-miR terminal loop in concert with its cofactors TRBP (also known as Loqs in *Drosophila*) and PACT. In this process, Dicer binds to the pre-miR 2 nt 3′ overhang and cuts two helical turns (~22 nt) away to produce a double-stranded RNA with 3′ overhangs of 2 nt at both ends. TRBP and PACT regulate Dicer’s substrate recognition and RNA processing power but are not essential for Dicer’s slicing activity [27–33]. After cleavage, the strand with the 5′ terminus that has less stable base-pairing (the “guide strand”) is transferred onto an Argonaute (Ago) protein, which is part of a poorly defined multi-protein miR-induced silencing complex (miRISC) that includes Dicer, TRBP and TNRC6 (also known as GW182) whereas the other strand (the “passenger” or “star strand”) is degraded [34–37]. Additional features on the miR duplex may also play a role in the strand selection process and there are several miRs where both strands are incorporated, to varying degrees, onto Ago proteins [38–41]. Once in place, the miR nucleotide sequence serves as a guide for RNA interference (RNAi) based on the partial complementarity with the various RNA substrates, a process which is largely attained by random diffusion of miRISC into the cytosol [42]. TNRC6 proteins are essential for RNAi as they interact with poly(A)-binding protein (PABP) and the PAN2–PAN3 and CCR4-NOT deadenylase complexes to induce translation repression, deadenylation and decay of the mRNA targets [43–45].

## 3. Efficacy of miR Repression

**Figure 2:**
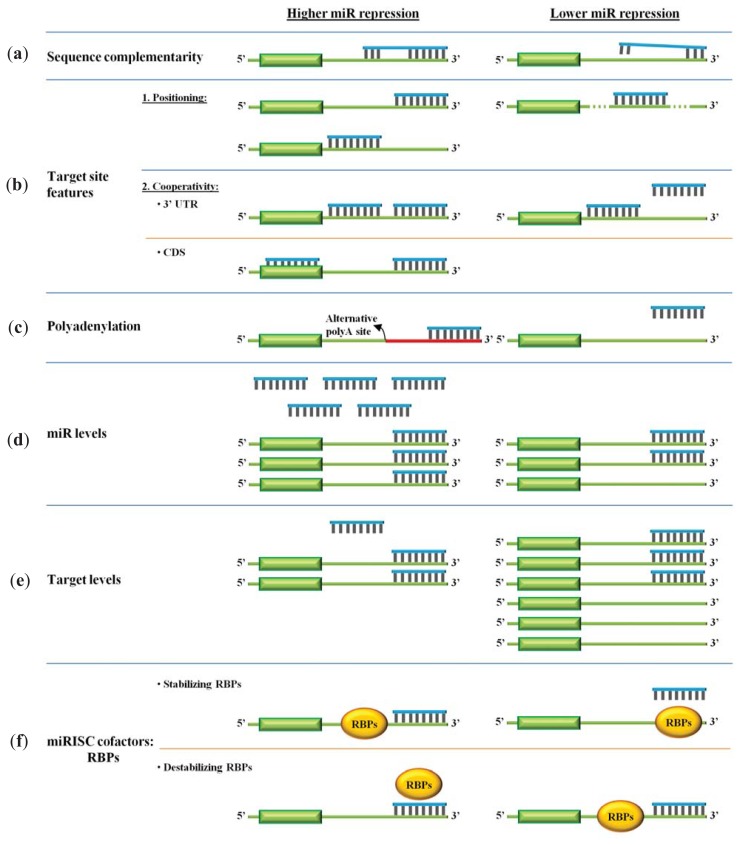
miR repression determinants. Multiple factors determine repression effectiveness of miRs. These include: (**a**) Sequence complementarity at positions 2–7 or 2–8 of the 5′ end of the mature miR; (**b**) Target site features: binding site location near the edges of 3′ UTR or multiple binding sites for miRs; (**c**) Alternative cleavage and polyadenylation to maintain miR binding sites; (**d**) Relatively high miR levels; (**e**) Relatively low target levels; and (**f**) Presence of stabilizing and absence of destabilizing RNA binding protein sites.

Despite a wealth of genome-wide and biochemical data on the role of miRs in the regulation of their targets, we do not yet have a clear understanding of the factors that determine which mRNAs will be targeted by miRs or by which mechanism individual mRNAs will be silenced, that is, translation repression or mRNA destabilization. Likely, this reflects on the vast repertoire of context-specific determinants that modulate miR-target interactions (Figure 2).

### 3.1. The miR Nucleotide Sequence

Large-scale transcriptomic and proteomic studies have revealed that the primary determinant for miR binding is perfect consecutive Watson-Crick base-pairing between the target RNA and the miR at positions 2–7 or 2–8 of the 5′ end of the mature miR, often denoted as the “seed” region [46–49]. This signature has been reaffirmed with crystallographic studies of ribonucleoprotein Ago-miR complexes showing that the seed region is organized in a helical conformation that exposes it to base-pair with the target RNA [50–52]. More recently, a genome-wide analysis of Ago sites in murine brain revealed a variant of this target recognition pattern through a single bulged nucleotide in the middle of the 2–7 seed. These bulged sites, that likely yield overall lower repression, are evolutionarily conserved and comprise over 15% of all Ago-miR interactions, thus, expanding significantly the number of potential miR regulatory sites [53]. Despite the aforementioned basic features, a “seed” is neither necessary nor sufficient for target silencing. It has been shown that miR target sites can often tolerate G:U wobble base pairs within the seed region [54,55] and extensive base pairing at the 3′ end of the miR may offset missing complementarity at the seed region [46,56]. Moreover, centered sites have also been reported showing 11–12 contiguous nt base-pairing to the central region of the miR without pairing to either end [57]. To add to this repertoire, other studies report efficient silencing from sites that do not fit to any of the above patterns, seemingly appearing random [58,59], and even sites with extensive 5′ complementarity can be inactive when tested in reporter constructs [60].

### 3.2. Target Site Features

Considerable progress has been made to identify additional features that could predict target regulation with more precision. Grimson *et al.* have reported that local sequence context, such as AU-rich nucleotide composition near the target site, proximity to sites for co-expressed miRs, proximity to residues pairing to miR nt 13–16, and positioning away from the center of long 3′ UTRs can all promote efficient miR repression of targets [61]. With respect to these findings, several different studies have reaffirmed that multiple miR sites in the same 3′ UTR can potentiate the degree of translational repression. They reported that optimal downregulation is obtained when two sites are closely positioned, usually between 13 and 35 nt apart [62,63]. However, target sites spaced at substantially longer distances may still cooperate to lower the expression of proteins [64,65]. In this context, miR cooperativity is defined as the positive interaction of two or more individual miRs or one individual miR acting on multiple target sites on the same 3′ UTR for target regulation. Recently, it was estimated that the miR site density of brain synaptic mRNAs is twice higher than that of the rest of cellular mRNAs, indicating that miR cooperativity may be a prevalent mechanism for physiological processes that require precise control, such as synaptic transmission [65].

An additional feature that has also emerged is that miR target sites tend to be less evolutionary preserved in the first ~15 nt downstream of the stop codon, presumably, to avoid being in the path of the translational machinery that could displace the miRISC complex [61]. However, both computational and biochemical approaches have later identified that nearly half of miR sites are located in open reading frame (ORF) sequences [66–70]. Experimental analysis indicated that the sites in coding regions and to a lesser extent 5′ UTRs can confer miR repression, albeit at lower levels than 3′ UTRs [71,72]. Recently, it was reported that coding region-located sites induce more rapid reduction in mRNA translation than 3′ UTR-located sites in a process that does not involve mRNA degradation, however, the effect may only be transient. The authors elaborate that this type of response may be suited for the regulation of cell cycle proteins [73]. Further, there are several families of paralogous genes that contain multiple repeat sequences in their coding regions, arisen through evolutionary duplications, that are miR targets [74]. Like for miR cooperativity in 3′ UTRs, it was shown that miR sites in the coding region potentiate the repression activity of miRs acting on 3′ UTR [75].

To add a twist to miR regulation, it has been reported that individual miRs may also display distinct preference for binding to different regions of an mRNA. For instance, neuronal miR-124 seed sequences are preferentially located in the 3′ UTR, while miR-107 seed sequences are enriched in the coding region of the mRNAs. Further, mRNA targets of neuronal miR-128 and miR-320 are less enriched for 6-mer seed sequences than miR-124 and mir-107 [76]. The reason for these differences is unknown but they, evidently, enrich the heterogeneity of miR-mediated repression.

### 3.3. Target Accessibility and Polyadenylation

Another important determinant of efficient silencing is target RNA folding with several reports indicating that miR sites are preferentially positioned in highly accessible/unstructured regions at the start and end of 3′ UTRs [61–79]. Experimentally, target sites in the middle of 3′ UTR have been found to be less efficient for RNAi regulation [80] while those positioned near the end of 3′ UTRs are associated with highest repression [62].

Another contributing factor is the length of the 3′ UTR. Approximately, half of human genes undergo alternative cleavage and polyadenylation (pA) to generate transcripts with variable 3′ UTR lengths [53]. Given that 3′ UTRs are the main targets of miRs, alternative pA is expected to modify target RNA translation. Consequently, a close connection between gene transcription and pA site choice has been demonstrated, in which highly expressed genes contain shorter 3′ UTRs while transcripts that are expressed in lower levels are associated with longer 3′ UTR isoforms [81]. Along this, higher gene expression is tightly linked to cell division where short 3′ UTR isoforms with fewer miR sites are abundant in proliferating cells [82]. In contrast, differentiated cells possess longer 3′ UTRs [81]. A noteworthy consequence of alternative splicing was observed in transformed cells where the loss of miR target sites by pA contributed to oncogene activation without any apparent mutagenesis [82].

### 3.4. miR and Target RNA Levels

An additional critical determinant for miR repression effectiveness is the cellular concentrations of (a) the target RNA, (b) the miR and (c) the miRISC complex. miRs that have multiple targets and are not highly expressed are expected to downregulate individual target genes to a lesser extent than those with a lower number of targets. Similarly, highly abundant target transcripts, that may act as decoys, dilute the effect of miRs under differential conditions [83–85] and this effect is more pronounced when the decoys are capable of perfect base-pairing with the miR [86]. Along these lines, it has been observed that lower levels of a miR may fail to regulate its target mRNA, however, it retains the ability to promote inhibition in conjunction with another miR, indicating that cooperative silencing requires lower concentration of miRs [65]. Going beyond, it is predicted that imbalances in the relative concentrations of miRs and their gene targets may exaggerate or compensate for sequence mismatches between miR and target RNA pairs. miRISC stability has emerged as an additional level at which miR activity can be controlled. Specifically, LIN41, an E3 ubiquitin ligase, has been shown to target Ago2 for ubiquitination and proteasome degradation. Because Ago proteins are limiting factors for the activity of the miRISC complex, alterations in the levels of LIN41 result in global attenuation of miR-mediated repression [87].

### 3.5. RNA Binding Proteins

RNA binding proteins (RBPs) regulate key aspects of gene expression including pre-mRNA splicing, nuclear-cytosolic shuttling, cytosolic transport and storage, local translation and turnover. Although most RBPs have housekeeping functions, a subset of RBPs controls the expression of specific labile mRNAs by binding to U- and AU-rich elements (AREs) on either 5′ UTR or 3′ UTR. These include HuR, TIAR, TIA-1, AUF1, TTP and KSRP, collectively known as translation and turnover regulatory (TTR)-RBPs. They primarily modulate mRNA levels in response to external stimuli and have been shown to influence all aspects of cellular activities that include proliferation and differentiation [88]. Recently, a link between RBPs and miRs has emerged. Initially, it has been observed that destabilization mediated by a transfected miR is generally attenuated by the presence of destabilizing AU-rich motifs and augmented by stabilizing U-rich motifs, the binding sites of TTR-RBPs [89,90]. Subsequently, transcriptome-wide analysis for the best characterized ubiquitous RBP, HuR revealed that most miR sites were found in the immediate vicinity of HuR sites [91,92] (reviewed in [93]). The authors elaborated that where miR and HuR sites overlapped the transcripts were preferentially regulated by HuR, but when they were not overlapping the transcripts were regulated by miR. Interestingly, HuR transcript is itself a direct target of miRs and of itself, and at the same time, directly regulates stability and/or maturation of other miRs pointing to the vast repertoire of the different regulatory loops [91–96].

## 4. Availability of miRs

**Figure 3:**
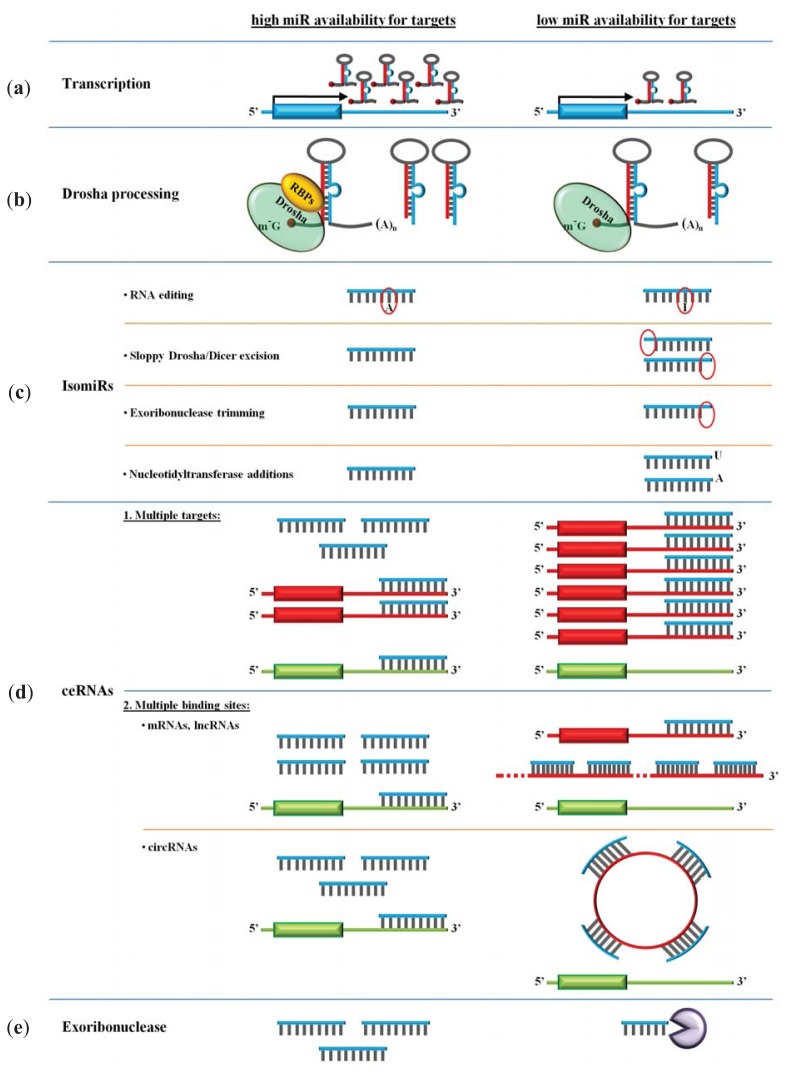
miR availability determinants. Multiple factors determine availability of miRs. These include: (**a**) High transcription rates; (**b**) Enhanced Drosha processing; (**c**) Lower levels of isomiRs that result from RNA editing, sloppy Drosha/Dicer cleavage, exoribonuclease trimming and nucleotidyl transferase additions; (**d**) Lower levels of miR sequestering ceRNAs; and (**e**) Lower levels of exoribonucleases.

It has become increasingly evident that miR activity is determined not only by target site features but also miR levels, target abundance and the presence of multiple RNA decoys (Figure 3). It is the summation of all these inputs that ultimately shapes miR function.

### 4.1. Transcription

Most miRs are transcribed by RNA polymerase II (Pol II) and only few by RNA polymerase III (Pol III) [14–19,97]. Pol III-mediated transcription is usually restricted to housekeeping non-coding genes, such as tRNAs and snRNAs, that require ubiquitous expression under all conditions [98], whereas Pol II-mediated transcription permits tight control of expression during all types of regulatory conditions [99]. Nonetheless, there is evidence that the same promoter elements can be used by both polymerases in humans [100–102] and transcription factors can also regulate RNA Pol III activity to some degree [103]. Furthermore, whole genome analysis has revealed that miR promoters are, in general, very similar to protein-coding promoters containing proportionally similar levels of CpG islands, TATA boxes, TFIIB recognition elements (BRE) and initiators (Inr) [16].

The majority of miR genes are transcribed from introns (and to lesser extent exons) of protein-coding genes [17,20]. As a consequence, miRs, more often than not, are co-expressed with host genes [104]. Nevertheless, increasingly, there have been reports that showed that intragenic miRs could, independently, initiate transcription from own promoters [105–107]. It is now estimated that about a third of hosted miRs use their own promoters for more efficient and tailored transcription [14,16,108]. With respect to the miRs that are located in intergenic regions, these are often arranged in clusters that lead to one pri-miR being subsequently processed into several mature miRs [104]. Using microarray profiling, Baskerville and Bartel have proposed that miRs separated by <50 kb are typically derived from a common transcript [104]. Accordingly, the latest miRBase release (Release 20) groups human and murine miRs in 153 (containing 465 miRs) and 92 (containing 366 miRs) clusters, respectively, using a default of <10 kb inter-miR distance. Clusters provide an effective mechanism to express cooperative miRs, simultaneously. Many clusters contain representatives from different miR families that together regulate specific protein networks by co-targeting downstream mRNAs [109]. This provides another layer of coordinated system-wide regulation of gene output in cells [110].

### 4.2. Drosha Processing

Drosha has been shown to exert selectivity over its pri-miR substrates compared to other RNAs. The mechanism by which this is achieved differs between miRs. Thus far, microarray profiling has shown that subsets of miRs contain a Smad binding RNA sequence (R-SBE) within the stem region of the pri-miR that resembles the Smad binding element in DNA. Smad proteins bind to these motifs on the miRs with one (MH1) domain while another (MH2) domain binds p68, a protein that is integral part of the microprocessor complex in the nucleus and is known to induce Drosha processing [111,112]. Similarly, DNA damage induces p53 association with p68, promoting the processing of specific miRs that subsequently exert a tumor suppressor function via repression of c-myc [113]. A different mode of regulation is demonstrated by the RNA binding proteins KHSRP and hnRNPA1 that bind to specific single- and double- stranded segments on the pre-miR hairpin, respectively, inducing microprocessor complex cleavage. Importantly, this targeted processing of the pri-miR has been shown to uncouple the uniform expression levels of clustered miRs from the maturation efficiency of individual miRs [114–116].

### 4.3. miR Polymorphism and isomiRs

Computational predictions have strongly suggested that miRs may have shaped the evolution of their targets based on the fact that the conservation of predicted miR target sites in mRNAs is higher than that of other conserved 3′ UTR motifs [117]. Consequently, polymorphisms in miR sequences were presumed rare. Towards this, bioinformatic analysis has revealed that the density of single nucleotide polymorphisms (SNPs) in miRs is 4.5-times lower than in protein coding sequences [118] and from these polymorphisms, only 1/10 or less are located in the seed region [60,118–120]. As expected, miR SNPs in the seed region would ultimately result in the regulation of a completely different set of mRNA targets. An increasing number of epidemiological reports have now linked several of these miR SNPs to pathology and, in particular, cancer susceptibility. miR-146a-3p and miR-499-3p, for instance, have so far been associated with the largest variety of cancer pathologies affecting all organ systems (for review see [120,121]).

Recent advances in high-throughput small RNA sequencing technologies have revealed novel post-transcriptional processing mechanisms that increase mature miR sequence heterogeneity from single genomic locus in cells. It is estimated that as many as 90% of miRs are presented with some sort of modification mainly in the form of trimming and/or nucleotide addition in the 3′ terminus [122]. Thus far, four mechanisms that generate functionally distinct miR isoforms, annotated as isomiRs, have been identified [123]. These are RNA editing, inexact Drosha and Dicer processing, exonuclease ribonucleotide trimming and template-independent ribonucleotide addition.

RNA editing is a chemical alteration in the primary nucleotide sequence of double-stranded RNAs. The most common RNA editing modification involves the hydrolytic deamination of adenosine-to-inosine (A-to-I) catalyzed by the adenosine deaminase acting on RNA (ADAR) enzymes [124]. Because inosine preferentially base pairs with cytidine, this conversion is equivalent to an adenosine to guanosine change. Although earlier reports identified widespread A-to-I editing in pri-miRs, more recent studies have revealed that RNA editing is rather rare for mature miRs [125,126]. A comprehensive profiling of human RNA editome revealed only 44 edited miR sites of which 11 were in the seed region [127]. This indicates that miRs exhibit low frequency of editing and that the primary biological function of miR editing in animals is the regulation of the miR maturation pathway, rather than the specificity of miR targeting [125]. Nevertheless, editing of mature miRs at seed region, such as for the most thoroughly studied mir-376, resulted in changes in the targeting profile that subsequently altered biological function in a tissue-specific manner [126] promoting carcinogenesis [128]. For another miR, mir-142, pri-miR editing resulted in impaired Drosha processing and enhanced degradation by the specific I-U nuclease Tudor-SN [129]. Recently, the adenosine deaminase ADAR1 was shown to differentiate from its deaminase activity and participate in RNAi when in heteroduplex with Dicer. Hence, when in complex with Dicer, it increased the rate of pre-miR cleave and facilitated miRISC loading of mature miRs, while in homodimer form, it mediated RNA editing [130].

Multiple isomiRs with various 5′ and/or 3′ ends are thought to be the result of sloppy Drosha and Dicer excision [131,132]. More recently, mammalian TRBP and its *Drosophila* ortholog Loqs have been shown to fine-tune Dicer cleavage sites for a subset of miRs generating longer miR isoforms by one nucleotide at either 5′ or 3′ ends [133,134]. In addition, it was shown that the hairpin loop and stem structure of the pre-miR affected Dicer-TRBP processing with different sensitivity compared with Dicer alone. The authors proposed that TRBP might induce a Dicer conformational change influencing Dicer substrate specificity and kinetics [134].

Post-Dicer processing by exoribonucleases modulates 3′ shortening in miRs. Nibbler, a 3′ to 5′ exoribonuclease has been shown to trim Ago-bound miRs in *Drosophila*; depletion of Nibbler resulted in the loss of about a quarter of 3′ isomiRs; unexpectedly, Han *et al.* also found that miRs are frequently produced by Dicer as intermediates that are longer than ~22 nt, and are subsequently trimmed to appropriate size by exoribonucleases [129,135]. It remains to be seen whether similar mechanisms exist in mammals.

Besides nucleotide excisions, post-Dicer 3′ additions are widespread and conserved. These are mediated by several nucleotidyl transferases that catalyze the addition of ribonucleotides, most often adenine and uridine, to the ends of mature miR molecules [136,137]. Interestingly, these isomiRs are differentially expressed across development and different tissues. For instance, adenines are highly abundant in early *Drosophila* development, while a subset of miRs with uridines is expressed in adult tissues [138]. With respect to function, these 3′ ribonucleotide additions have been shown to alter (enhance or lower) miR stability in some cases [138,139] and/or effectiveness in others [140,141]. However, the authors concluded that these effects are likely to be restricted to only a small subset of isomiRs in animals [140]. Like for differentially expressed splice mRNA variants, several isomiRs with 3′ additions have been associated with human diseases. Thus far, significant alterations have been reported in cancer, Huntington’s disease and pre-eclampsia [122,142,143].

### 4.4. ceRNAs and miR Degrading Enzymes

Recently, a new model of post-transcriptional regulation has emerged in which RNA targets are not merely passive substrates of miR repression, but cross-talk with each other in distinct networks by competing for shared miRs. Such competing endogenous RNAs (ceRNAs) ultimately determine mature miR availability and function within cells (reviewed in [144,145]). This reverse reasoning compels a redefinition of the idea that miRs stand at the top of mRNA networks to regulate protein output, by considering that any RNA that shares same target sequences actively regulate each other and miRs through direct competition for miR binding. Initial reports that provided proof of principle to this concept have shown that exogenous overexpression of 3′ UTR sequences alone titrated cellular miR abundance and inactivated miR functions by freeing target mRNAs from repression [83,146,147]. Subsequently, it was shown that tenths of protein-coding mRNAs that share multiple miR target sites with dose-sensitive phosphatase and tensin homolog (PTEN) act as decoys to modulate PTEN levels [85,148]. An implication of these studies is that any RNA with miR target sites can potentially function as ceRNA. Thus, long non-coding RNAs (lncRNAs), due to their length, may be good candidates for sequestering miRs within cells. Hence, muscle-specific lncRNA, linc-MD1, was shown to sponge out two miRs to regulate the expression of transcription factors that activate muscle-specific gene expression [149]. Similarly, the PTENP1 pseudogene that is highly homologous to PTEN regulated cellular levels of PTEN (and the reverse) by sponging out common miRs [150]. Very recently, the repertoire of ceRNAs has been expanded by the identification of a new subclass of circular RNAs (circRNAs) [151,152]. Like other ceRNAs, these circRNAs serve as miR reservoirs. Distinctly, however, circRNAs may have multiple binding sites for specific miRs and therefore, are dedicated to sequestering particular miRs. Furthermore, being circulized, they possess enhanced stability by avoiding RNA exoribonuclease enzymes that act on 3′ and 5′ RNA ends and hence, maintain their effects for longer. An extreme case is characterized by human circRNA, ciRS-7, that harbors 74 mismatched mir-7 seed matches of which 63 are conserved in at least one other species [152]. This circRNA acts as a mir-7 sponge; it is resistant to miR-dependent destabilization and strongly suppresses miR-7 activity [151].

Exoribonucleases and the exosome have also been implicated in miR turnover. Using microarrays, Bail *et al*. have found that most miRs are remarkably stable (half-life over 8hrs), but some, including miR-382, were short-lived and were degraded to a modest extent (1.5-fold) by XRN1, a 5′ to 3′ exoribonuclease, and exosome, but not by XRN2 [153]. Moreover, overexpression of polynucleotide phosphorylase hPNPase(old-35), a 3′ to 5′ exoribonuclease, resulted in the downregulation of specific mature miRs in human melanoma cells without affecting their pri- or pre- miR levels [154].

## 5. Non-Canonical miR Activities

A relatively small number of studies have demonstrated that miRs can stimulate gene expression along their assigned repressive roles. These reports indicated that miR-mediated effects via gene promoters, extracellular receptors and 3′ or 5′ UTRs can be selective and controlled, ordained by either the miR sequence, associated proteins and/or cellular context.

### 5.1. Promoter Activation

Earlier studies have shown that exogenous application of small duplex RNAs, that are complementary to promoters, activate gene expression just like proteins and hormones, a phenomenon referred to as RNA activation (RNAa) [155,156]. Soon later, Dahiya’s group discovered mir-373 target sites in the promoters of e-cadherin and cold shock domain containing protein C2 (CSDC2). miR-373 overexpression readily induced transcription of these two genes and this concurrent induction required mir-373 target sites in both promoters [157]. Subsequently, they showed that mir-205 sites are present in the promoter of interleukin (IL) tumor suppressor genes IL-24 and IL-32 and, similar to mir-373, mir-205 induced gene expression [157,158].

### 5.2. Target Activation

Several reports have shown that miRs can induce translation by binding to 5′ or 3′ UTR. In the brain, a target sequence of mir-346 was found in the 5′ UTR of a splice variant of receptor-interacting protein 140 (RIP140). Gain- and loss- of-function studies established that mir-346 elevated RIP140 protein levels by facilitating association of its mRNA with the polysome fraction. Furthermore, the activity of the mir-346 did not require Ago2 indicating that other RNPs in complex with the miR or different RIP140 mRNA conformation induced by the miR mediated the effect [159]. In another study, mir-145 was shown to regulate smooth muscle cell fate and plasticity via upregulation of myocardin (Myocd). Myocd bears mir-145 sites in 3′ UTR and mir-145 expression specifically upregulated luciferase expression by 150-fold; at the same time other mir-145 targets were repressed. It remains to be seen whether miR-145 interferes with binding of a destabilizing RBP to 3′ UTR [160]. Along this, miR-466l, a miR discovered in mouse embryonic stem cells, upregulated IL-10 expression in TLR-triggered macrophages by antagonizing the RBP tristetraprolin (TTP)-mediated IL-10 mRNA degradation [161].

### 5.3. Receptors’ Ligands

Members of the Toll-like receptor (TLR) family, mouse TLR7 and human TLR8, expressed on dendritic cells and B lymphocytes, physiologically recognize and bind ~20 nt viral single-stranded RNAs leading to their activation [162,163]. Because miRs are secreted in exosomes and are of similar size, it was predicted that they may also serve as TLR7/8 ligands. Indeed, Fabbri *et al*. identified that the tumor-secreted mir-21 and mir-29a were ligands for TLR7/8 and were capable of triggering a TLR-mediated prometastatic inflammatory response [164].

## 6. Conclusions

Over the past years, significant advances have been made into understanding how miRs interact with their RNA targets, and several key features, such as base-pair complementarity, local context factors and de/stabilization signals have been identified and finely analyzed as a result. The ultimate goal of all these studies has been to predict miR function through the identification of their targets. More recent analyses, however, demonstrated that local mRNA determinants could only explain a fraction of the miR repression activity and system level factors such as isomiRs, RBPs, and ceRNAs have been brought into attention. The very recent discovery that miRs can both regulate and be regulated by their RNA targets has presented a completely new twist into understanding the role of miRs in development and disease. It remains to be seen how miRs and RNA targets communicate using the miR nt sequence as a “language” to deliver large-scale concerted instructions in cells.
